# Supplementary material for: The Drosophila ovary produces two waves of adult follicles and a previously unreported pupal wave that turns over
Source: Development. 2026 Aug 10;153(15):dev205668. doi: 10.1242/dev.205668 (PMC13535104; doi:10.1242/dev.205668)
Supplement: Supplementary file 1 [file develop-153-205668-s1.pdf]

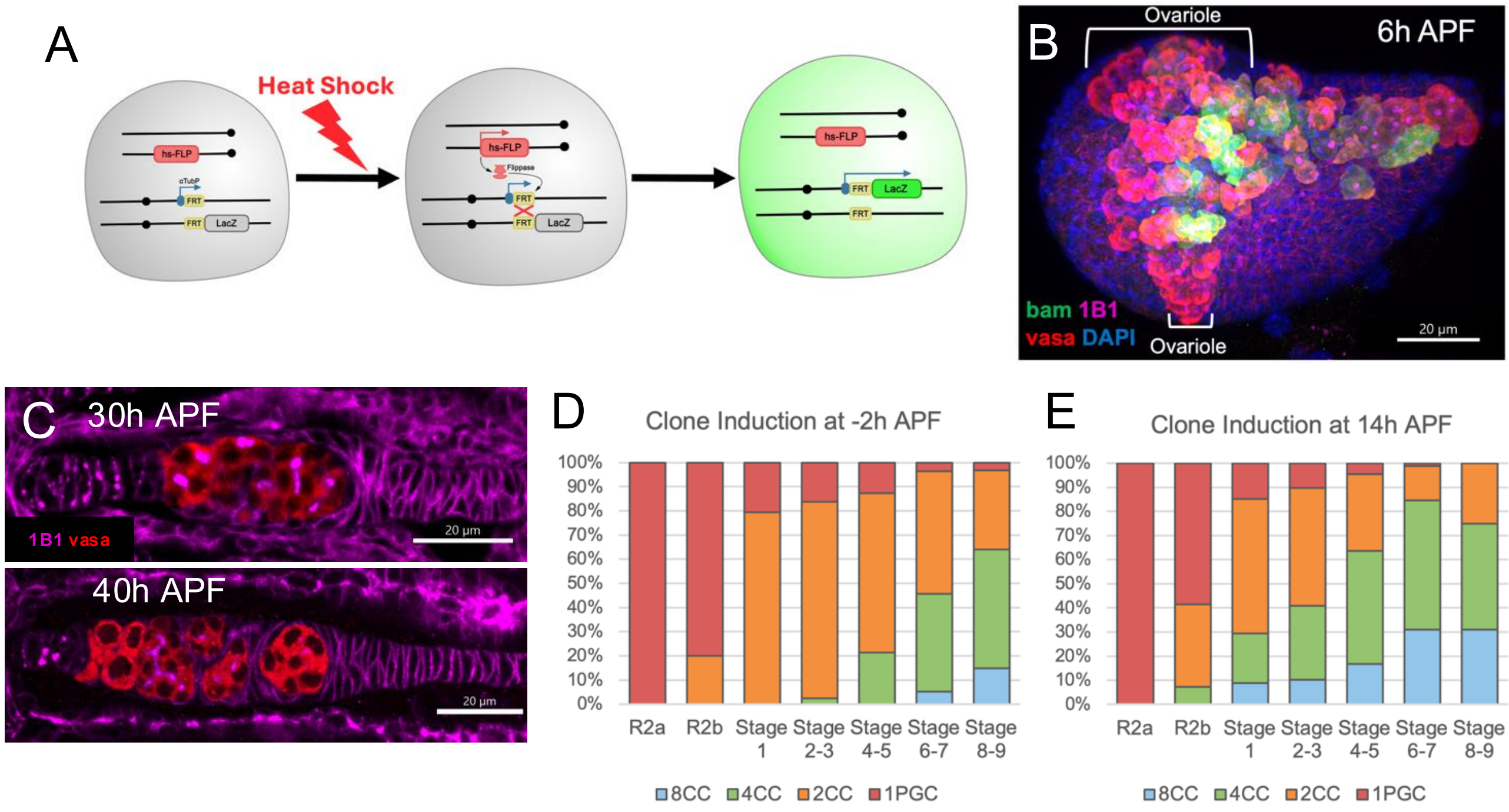

Fig. S1. Additional Information on Experimental Design and PGC biology

A. Schematic showing the detailed recombination process that gives rise to a lineage-labeled cell. **B.** An asymmetry in ovariole formation and PGC distribution exists in early pupal ovary, most prominent at 2h-6h APF and progressively becomes less evident over time. No significant asymmetry is observed by 18h APF. Ovarioles on the top of the image include more germ cells than the ovarioles on the bottom, as indicated by the brackets. PGCs posterior to the ovarioles are also unevenly distributed, more enriched on one side. 3D view shown in Movie S1. **C.** Example ovariole at 30h APF and 40h APF, both lacking the adult 2a/2b junction and R2b cyst morphology. **D-E.** Summary diagrams showing the labeling status of germ cells in wave 1.5 follicles following induction at -2h (F), or 14h (G).

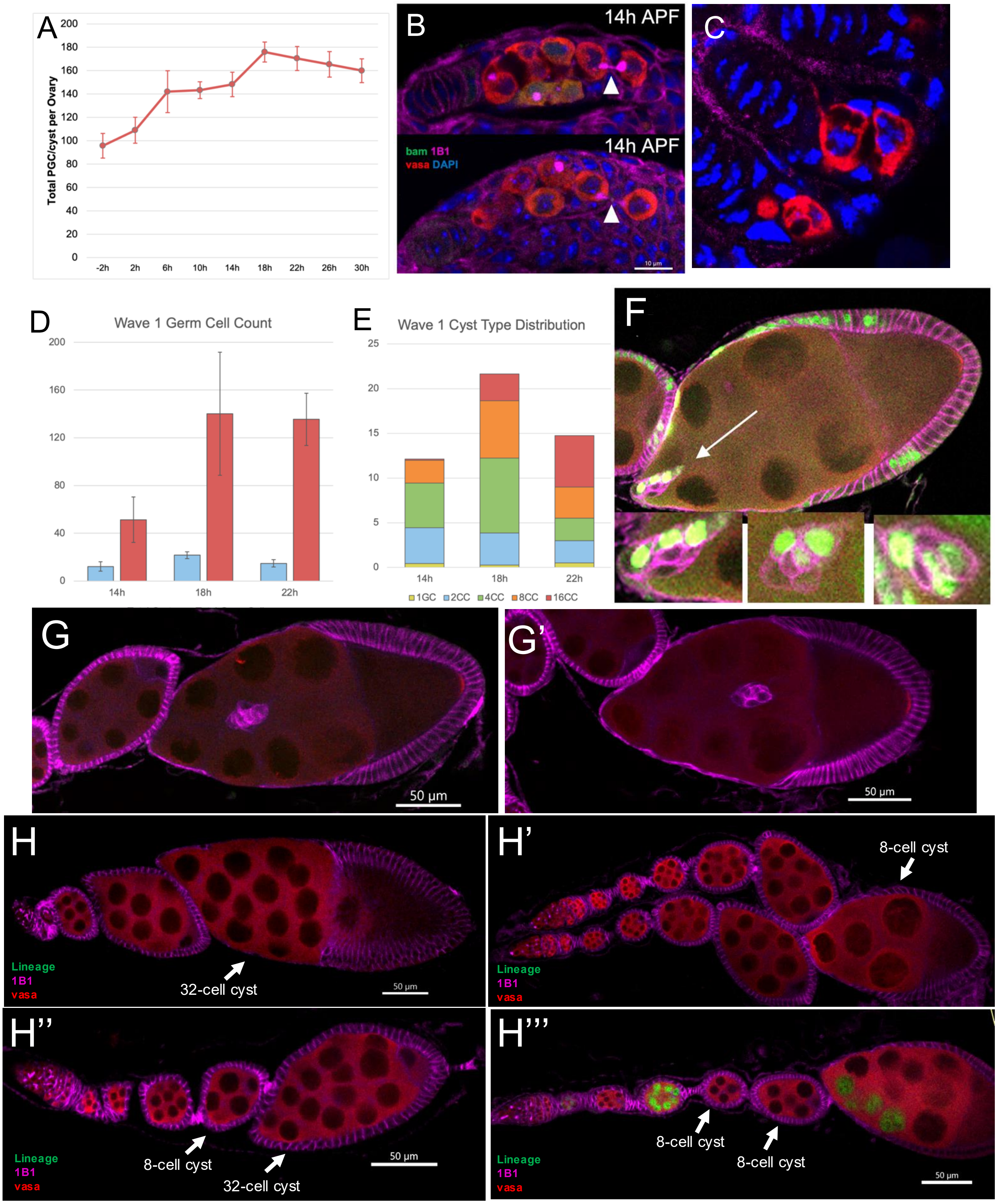

Development • Supplementary information

Fig. S2. Additional Quantifications of Wave 1 germ cells

A. Quantification of PGCs and cysts in the entire pupal ovary from -2h to 30h APF, showing the extent of PGC symmetric division and the dip caused by wave 1 loss starting at 22h APF. Quantification is based on counting the number of spectrosomes/fusomes, example counting process shown in Movie S2. **B.** Images showing the last rounds of PGC division take place among PGCs that give rise to wave 1.5 follicles at 14h APF. These are the PGCs that become included in the forming ovarioles but are too posterior to become GSCs. No more such divisions are observed at any time point later than 14h APF. **C.** A zoomed-out image of Figure 3N', showing that the single germ cells with vacuole inclusion are indeed GSCs next to the terminal filament, appearing as stacks of DAPI+ flat cells to the left side of the image. **D.** Quantification of wave 1 germ cells (red) and cysts (blue) from 14h to 22h APF. Germ cell count stays the same while cyst number drops from 18h to 22h APF, due to the loss of wave 1 cysts initiating at 22h while the overall number being compensated by continuous cyst division. **E.** Zoomed-in display of the blue "Total Cysts" bars in S2A, detailing the relative proportion of cysts at different sizes in the total population. **F.** A final wave 1.5 follicle now at stage 9 that was lineage labeled at 14h and contains a follicle cell clone which extends to the previous follicle. The border cell cluster at the anterior is mosaic (magnified in the box below) with 3 marked cells, and migrates slower than in adults toward the posterior. Other examples show the large size of the polar cells in these clusters and the presence of 3-4 labeled cells. **G-G'**. More examples showing polar cells migrate posteriorly at different speed than main body follicle cells, while in adults they migrate together. **H-H'''.** Ovarioles of newly eclosed flies showing abnormal cyst size of 8 cells and 32 cells in the last two wave 1.5 follicles, and the disproportionate polyploidization of several nurse cells.

Development • Supplementary information

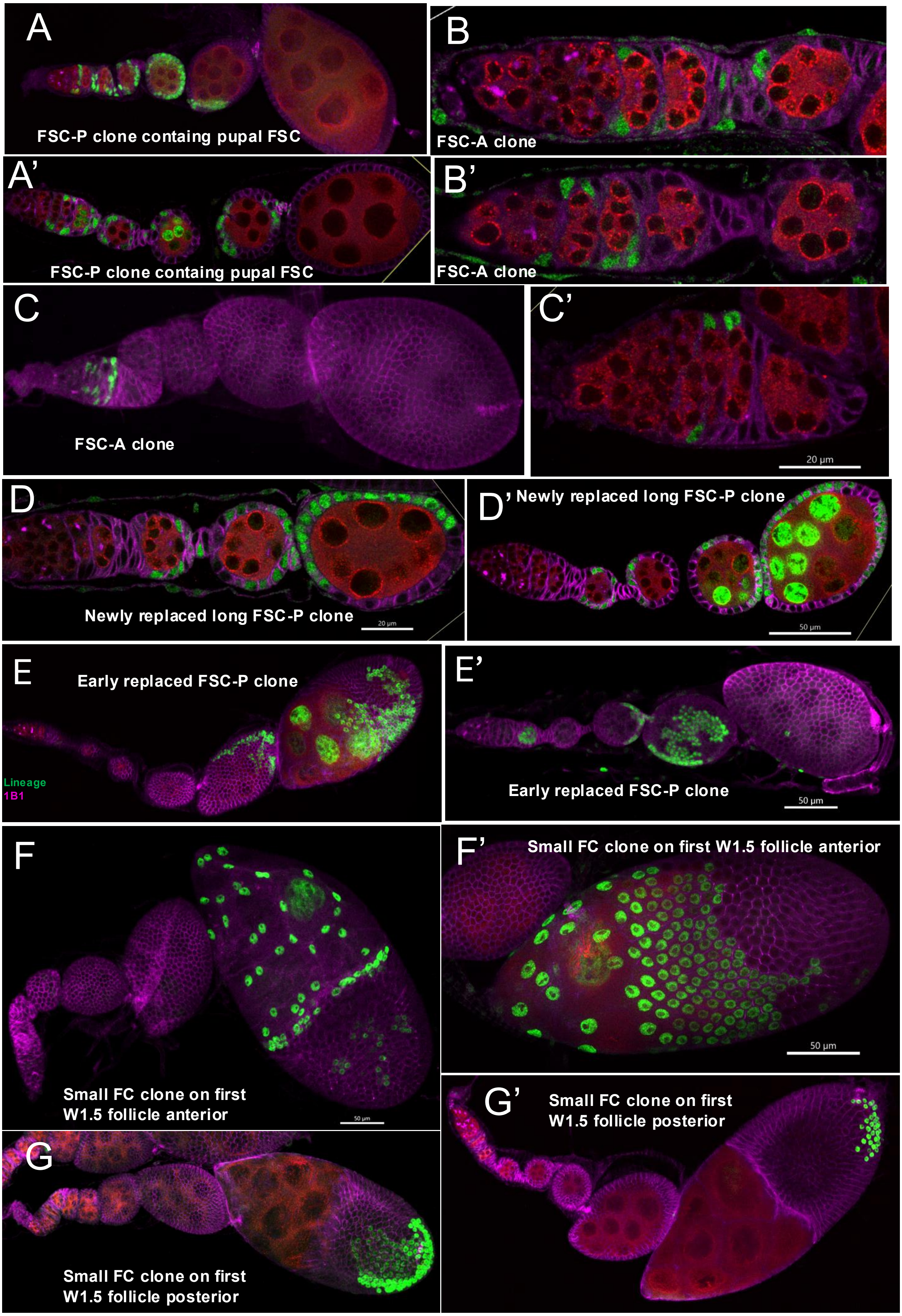
A
FSC-P clone containg pupal FSC
B
FSC-A clone
A'
FSC-P clone containg pupal FSC
B'
FSC-A clone
C
FSC-A clone
C'
20 µm
D
Newly replaced long FSC-P clone
20 µm
D' Newly replaced long FSC-P clone
50 µm
E
Early replaced FSC-P clone
Lineage
1B1
E'
Early replaced FSC-P clone
50 µm
F
Small FC clone on first
W1.5 follicle anterior
50 µm
F'
Small FC clone on first W1.5 follicle anterior
50 µm
G
Small FC clone on first
W1.5 follicle posterior
G'
Small FC clone on first
W1.5 follicle posterior

Fig. S3. Additional examples of FSC clones

A-A'. Ovarioles with long FSC-P clones that retained an active pupal FSC. **B-C.** Examples of FSC-A clones. C' is the magnified germarium region of the ovariole in C. **D-D'.** Ovarioles with long FSC-P clones that do not contain an active pupal FSC, replaced by an unlabeled FSC. **E-E'.** Short FSC-P clones on the older wave 1.5 follicles that had an early FSC loss. **F-F'.** The smallest follicle cell clones that only cover the anterior portion of the most mature wave 1.5 follicle, complementing the posterior patch pattern shown in G-G'. **G-G'.** Small patches of follicle cell clone that only cover the most mature wave 1.5 follicles, most likely originating from the basal stalk cells as previously reported (Reilein et al. 2021).

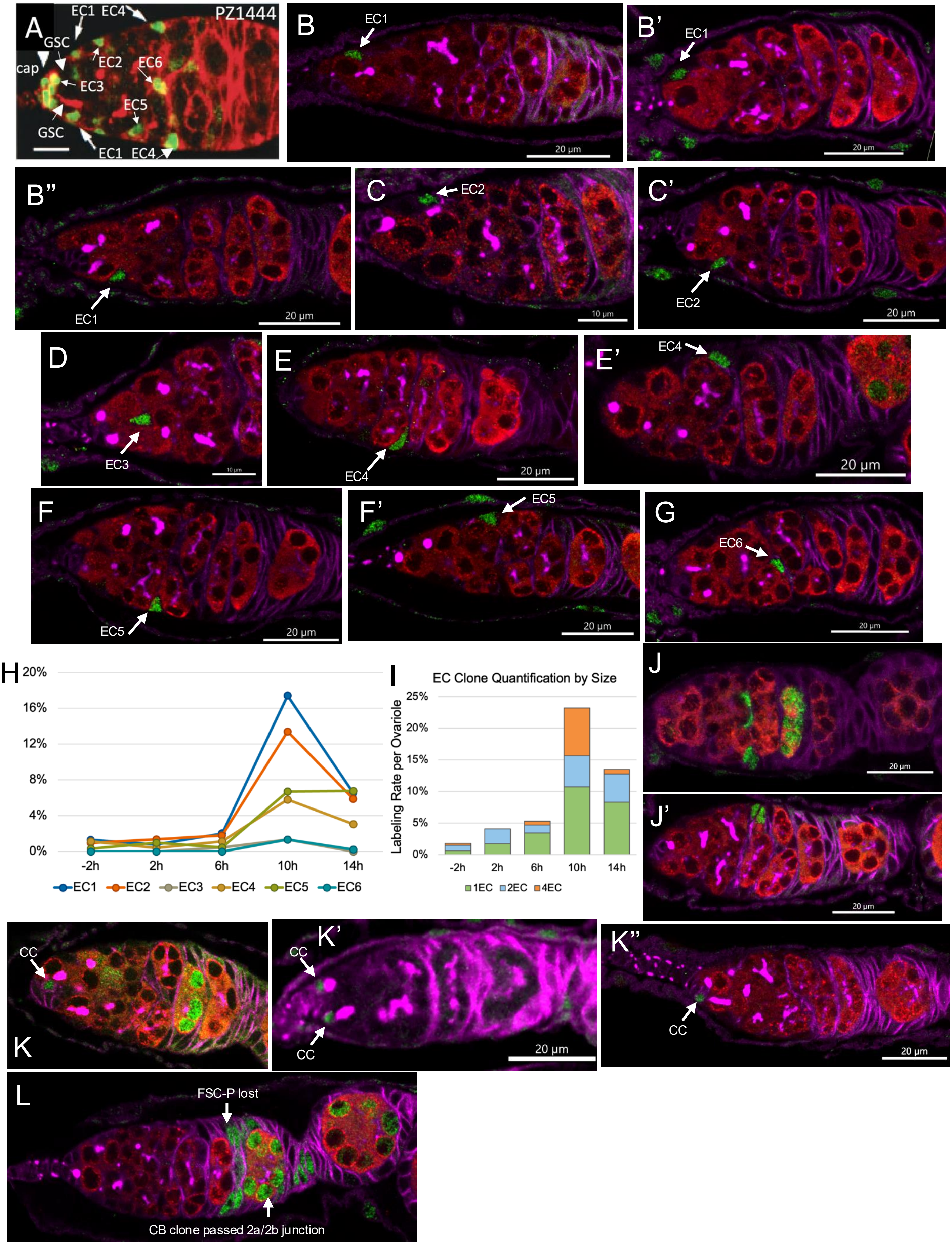

Development • Supplementary information

Fig. S4. Lineage labeled escort cell (EC) clones.

A. A germarium stained to mark the ECs using the enhancer trap line PZ1444. Two GSCs (unlabeled) are seen at the front associated with cap cells (arrowhead). In addition, 8 specific ECs are labeled and indicated with arrowheads and assigned corresponding names. B-L show germaria following clonal marking to identify EC-specific clones. **B-B''.** Example germaria each with one labeled EC1 (arrows). **C-C'.** Germaria with single EC2 clone. **D.** Germarium with an EC3 clone. **E-E'.** Germaria with single EC5 clone. **F-F'.** Examples of EC6 clone, immediately next to FSCs. **G.** An EC6 clone in the middle of the germarium at 2a/2b junction. **H.** Labeling frequency of EC clones based on the number of ECs each clone contains. **I.** Frequency of ovarioles with clones of ECs by type (EC1-6). **J.** Two adjacent labeled ECs, E5 and E4 are located directly adjacent to an unlabeled FSC. **J'.** An ovariole containing 4 ECs that are close to the 2a/2b junction. **K-K''.** Examples of cap cell clones. K' contains 2 labeled cap cells. **L.** An ovariole containing both a CB clone and a FSC-P clone that has lost its active pupal FSC, showing FSC-P loss happened slightly after the CB clone passed by 2a/2b junction.

Development • Supplementary information

Table S1. Germ cell clone quantification. The table on top records the number of germ cell clones at different sizes, stages and HS time points, with the total number of ovarioles in the first column. The bottom table shows lineage labeling frequency (number of clones/number of total ovarioles) of all clone types in the top table. The first header row indicates the location of recorded germline clones, and the second header row indicates the size of such clones (e.g. 4-8 represents tally of 4-cell and 8-cell germline clones, etc.) For germ cells, only one replicate group is fully quantified due to low level of variation (n=346, 303, 129, 224, 164 ovarioles for each time point, respectively).

		GSC	R2a	R2b		Stage 1		Stage 2-3		Stage 4-5		Stage 6-7		Stage 8-9		Stage 10A	
	**Ovarioles**	**16**	**16**	**4-8**	**16**	**4-8**	**16**	**2-8**	**16**	**2-8**	**16**	**1-8**	**16**	**1-8**	**16**	**1-8**	**16**
**-2h**	**341**	70	13	6	33	24	9	37	6	34	5	59	4	60	1	2	0
**2h**	**303**	59	34	3	51	25	8	51	11	65	7	65	3	81	0	67	0
**6h**	**124**	36	3	2	15	11	2	15	2	28	1	21	1	48	0	49	0
**10h**	**223**	64	14	18	33	37	4	51	5	56	4	88	0	116	1	24	0
**14h**	**160**	45	13	17	22	27	4	39	5	56	3	85	1	107	0	1	0
**Total**	**1151**	**274**	**77**	**46**	**154**	**124**	**27**	**193**	**29**	**239**	**20**	**318**	**9**	**412**	**2**	**143**	**0**

	GSC	R2a	R2b		Stage 1		Stage 2-3		Stage 4-5		Stage 6-7		Stage 8-9		Stage 10A	
	**16**	**16**	**4-8**	**16**	**4-8**	**16**	**2-8**	**16**	**2-8**	**16**	**1-8**	**16**	**1-8**	**16**	**1-8**	**16**
**-2h**	18.2%	3.8%	1.7%	9.5%	6.9%	2.6%	10.7%	1.7%	9.8%	1.4%	17.1%	1.2%	17.3%	0.3%	0.6%	0%
**2h**	18.0%	11.2%	1.0%	16.8%	8.3%	2.6%	16.8%	3.6%	21.5%	2.3%	21.5%	1.0%	26.7%	0.0%	22.1%	0%
**6h**	17.8%	2.3%	1.6%	11.6%	8.5%	1.6%	11.6%	1.6%	21.7%	0.8%	16.3%	0.8%	37.2%	0%	38.0%	0%
**10h**	28.9%	6.3%	8.0%	14.7%	16.5%	1.8%	22.8%	2.2%	25.0%	1.8%	39.3%	0%	51.8%	0.4%	10.7%	0%
**14h**	28.3%	7.9%	10.4%	13.4%	16.5%	2.4%	23.8%	3.0%	34.1%	1.8%	51.8%	0.6%	65.2%	0%	0.6%	0%

Development • Supplementary information

Table S2. Somatic cell clone quantification. The top table is the raw count of each type of somatic cell clone at each HS time point, the bottom table shows the labeling rates in percentage of each type of somatic cell clone (number of ovarioles with such clone over the total number of ovarioles). The top table shows the count of each replicate (e.g. 2h-1 and 2h-2 are two replicates of the 2h HS time point), while the bottom table shows the average labeling rate of each time point. 1CC: clone of 1 cap cell; 2CC: clone of 2 cap cells; 1EC: clone of 1 EC; 2EC: clone of 2 ECs; EC-M: clone with 4 or more ECs (87% are 4 EC clones); FSC-A: number of short adult FSC clones that correspond to wave 2 follicles (see text); FSC-P (with FSC): number of long FSC clones that contains an active pupal FSC (see text); long FSC-P (without FSC): number of pupal FSC clones covering multiple consecutive wave 1.5 follicles that are missing an active FSC (see text); short FSC-P (without FSC): number of short pupal FSC clones covering only 1 or 2 of the most mature wave 1.5 follicles (see text). n=621, 517, 449, 224, 459 total scored ovarioles for each time point, respectively.

	Ovarioles	1CC	2CC	1EC	2EC	EC-M	FSC-A	FSC-P (with FSC)	Long FSC-P (without FSC)	Short FSC-P (without FSC)
-2h-1	**341**			2	2	1	6	5	5	13
-2h-2	**275**	1		2	3	1	1	2	2	6
2h-1	**303**	4	1	5			7	2		6
2h-2	**214**	3		4	5		7	1	1	7
6h-1	**124**	1		4			5	1		13
6h-2	**320**	5	1	12	4	2	7	2	3	16
10h-1	**223**	19	7	24	11	17	17	10	6	52
14h-1	**160**	10	1	10	2		11	1	11	32
14h-2	**111**	4		7	5	1	6	6	5	9
14h-3	**184**	9	1	23	14	1	13	7	7	29

	1CC	2CC	1EC	2EC	EC-M	FSC-A	FSC-P (with FSC)	Long FSC-P (without FSC)	Short FSC-P (without FSC)
-2h	0.36%	0%	0.65%	0.83%	0.33%	1.05%	1.09%	1.09%	2.97%
2h	1.36%	0.33%	1.76%	2.34%	0%	2.79%	0.56%	0.47%	2.63%
6h	1.17%	0.31%	3.43%	1.25%	0.63%	3.03%	0.70%	0.94%	7.54%
10h	8.48%	3.13%	10.71%	4.91%	7.59%	7.59%	4.46%	2.68%	23.21%
14h	4.86%	0.58%	8.30%	4.44%	0.72%	6.39%	3.27%	5.01%	14.46%

Development • Supplementary information

Table S3. Raw data recording the location and size of each individual observed clone. For interpretation please view the first legends tab inside the excel file.

Available for download at
https://journals.biologists.com/dev/article-lookup/doi/10.1242/dev.205668#supplementary-data

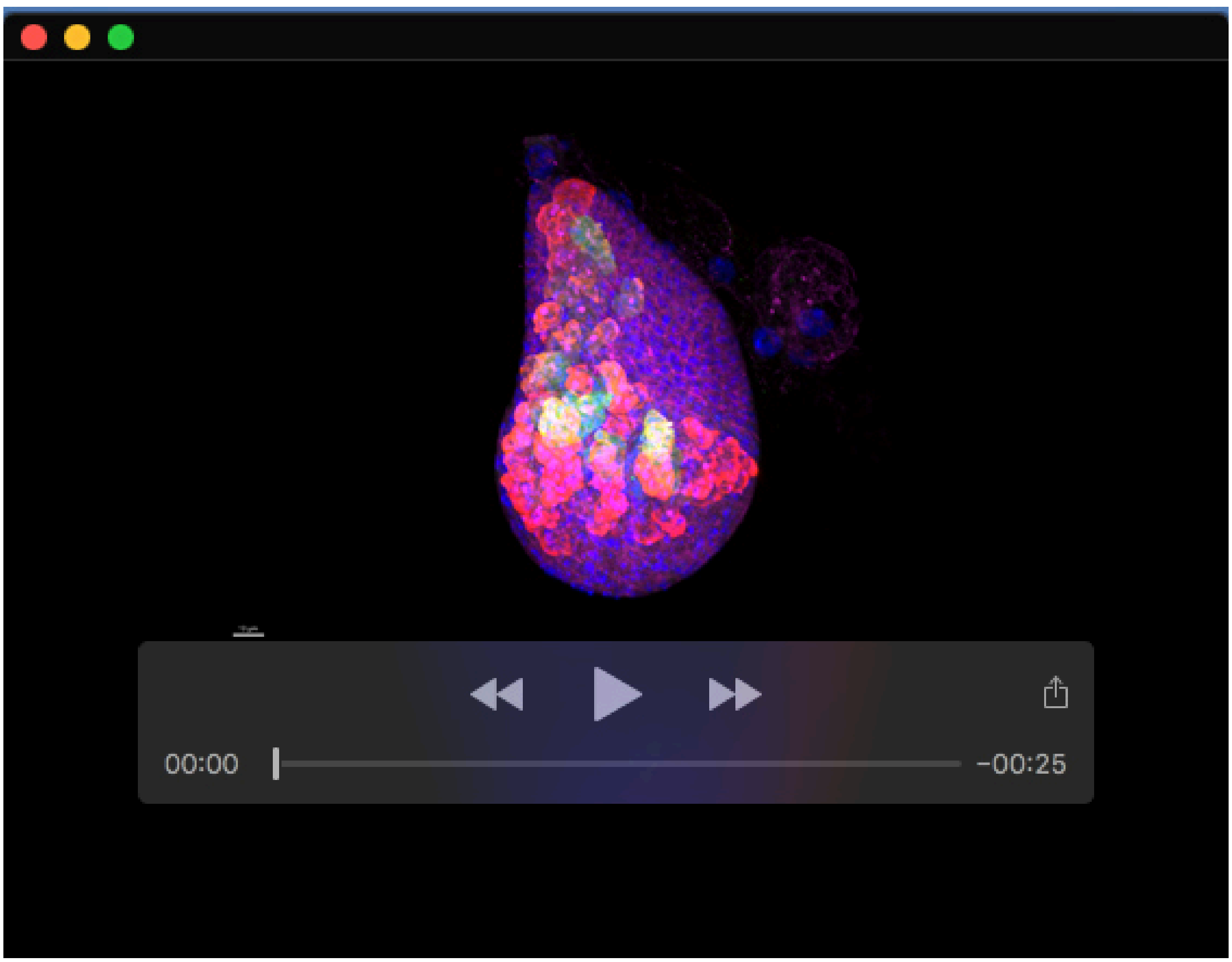

Movie 1. Rotation of a 6h APF ovary showing the asymmetry of PGC distribution along a dorsal-ventral axis.

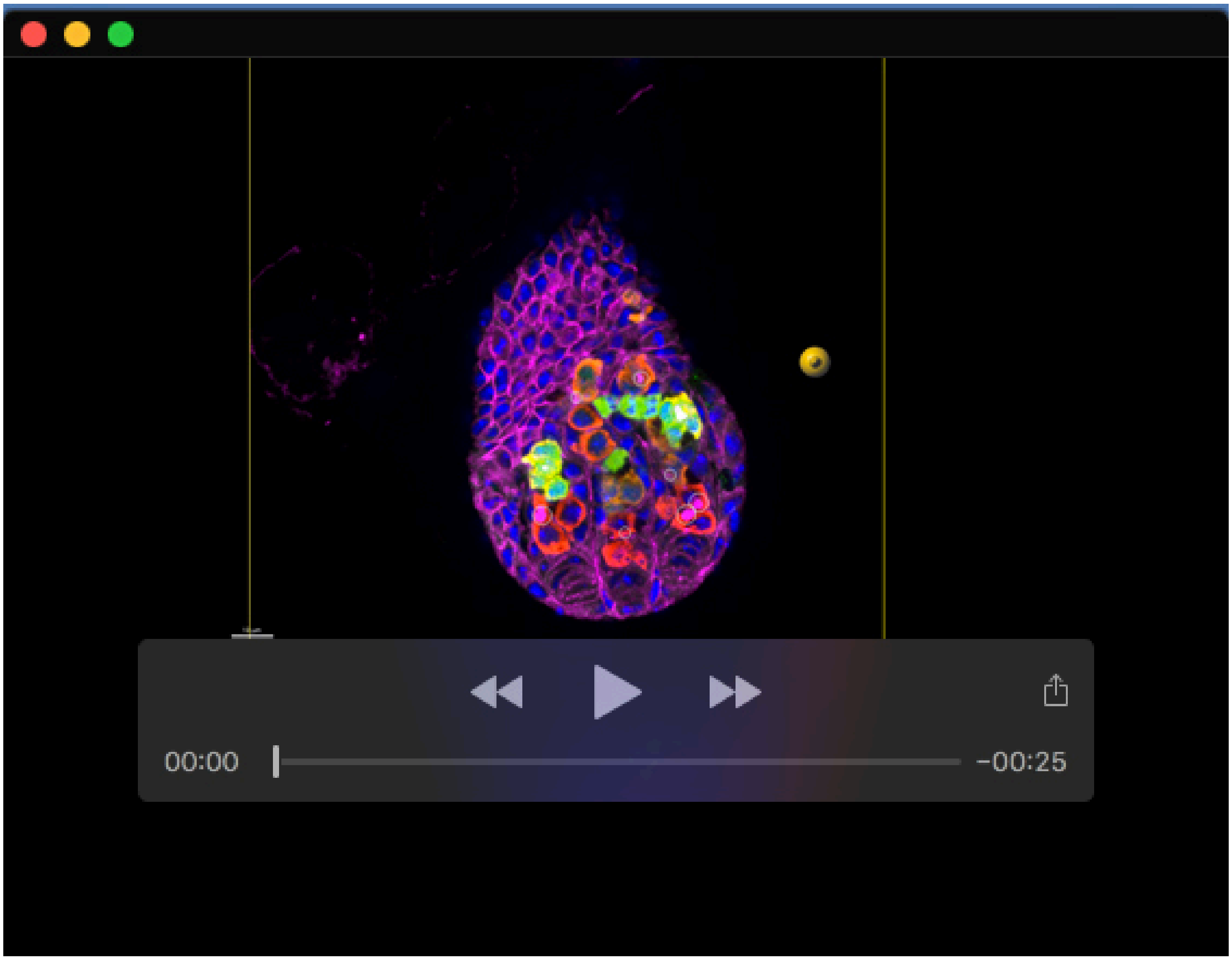

Movie 2. Example process of PGC counting in the pupal ovary. Spectrosomes/fusomes shown as magenta dots stained by Hts/1B1 are circled, as they are counted in Imaris using the Spot function.

Development • Supplementary information

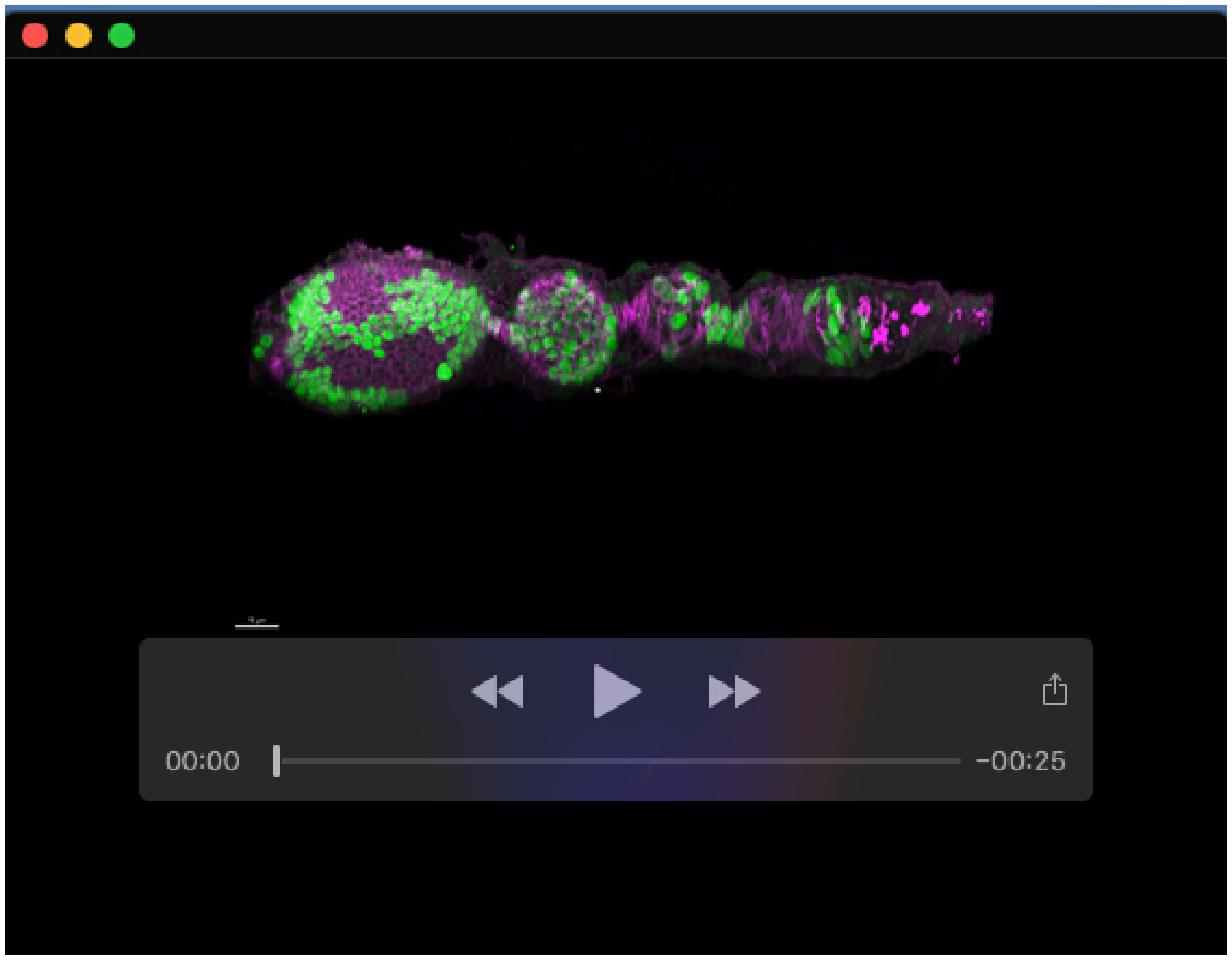

Movie 3. Examples showing how the quantification of follicle cell coverage in Figure 5K-L was done in Imaris in 3D reconstruction. Movie 3 shows an FSC-L clone.

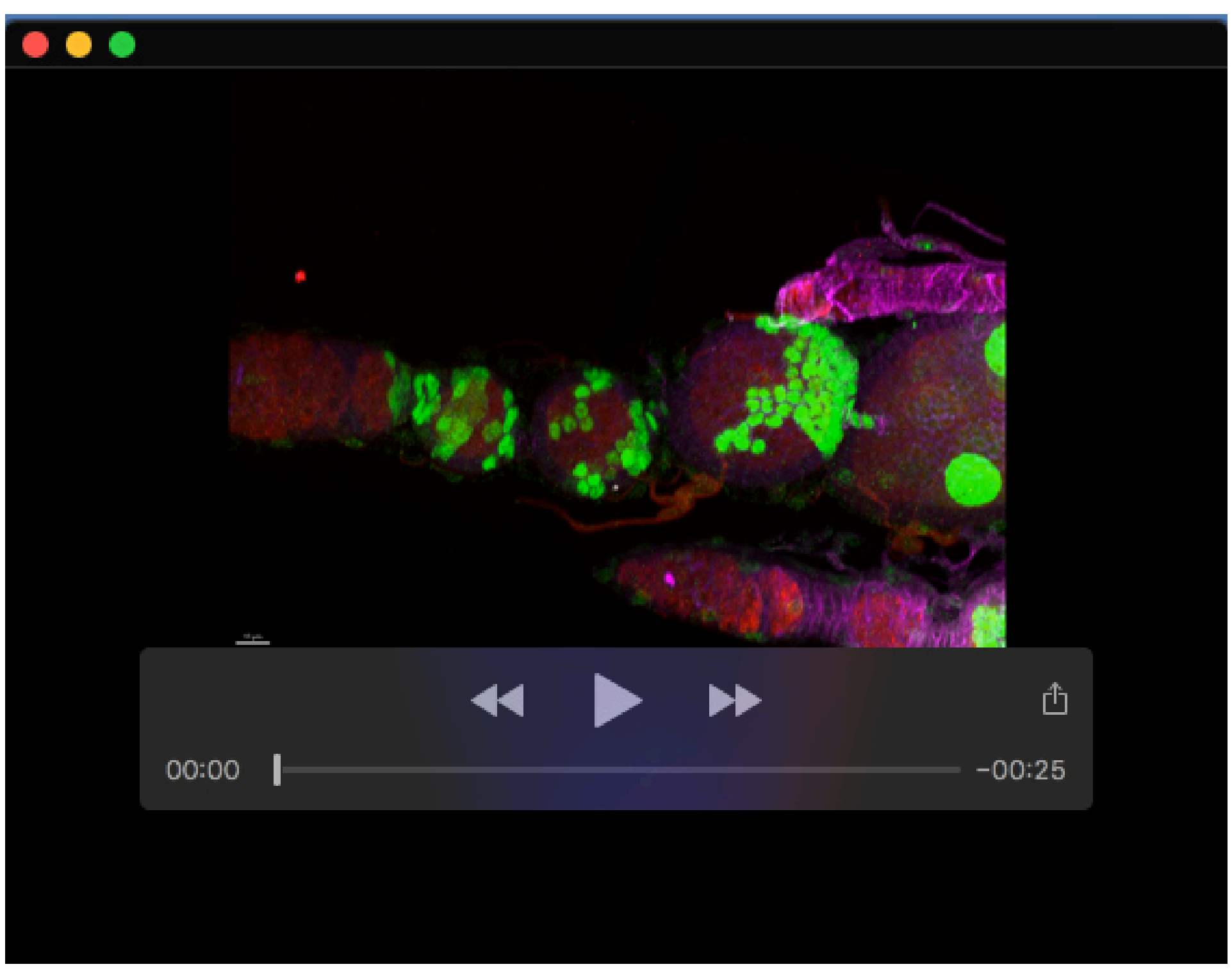

Movie 4. Examples showing how the quantification of follicle cell coverage in Figure 5K-L was done in Imaris in 3D reconstruction. Movie 4 shows an FSC-M clone.
