# Supplementary material for: The Drosophila ovary produces two waves of adult follicles and a previously unreported pupal wave that turns over
Source: Development. 2026 Aug 10;153(15):dev205668. doi: 10.1242/dev.205668 (PMC13535104; doi:10.1242/dev.205668)
Supplement: Supplementary file 2 [file develop-153-205668_review_history.pdf]

The *Drosophila* ovary produces two waves of adult follicles and a previously unreported pupal wave that turns over

Yunpeng Wayne Fu and Allan C. Spradling

DOI: 10.1242/dev.205668

Editor: Swathi Arur

Review timeline

Original submission:	17 March 2026
Editorial decision:	20 April 2026
First revision received:	1 June 2026
Editorial decision:	22 June 2026
Second revision received:	24 June 2026
Accepted:	25 June 2026

Original submission

First decision letter

MS ID#: dev.205668

MS TITLE: The Drosophila ovary produces two waves of adult follicles and a novel pupal wave that turns over

AUTHORS: Yunpeng Wayne Fu and Allan Spradling

Dear Dr Spradling,

I have now received all the referees' reports on the above manuscript, and have reached a decision. The referees' comments are appended below.

As you will see, the referees express considerable interest in your work, and provide recommendations to further improve the clarity and rigor of the data presented. If you are able to revise the manuscript along the lines suggested, some of which may involve additional experiments, I will be happy receive a revised version of the manuscript. Your revised paper will be re-reviewed by one or more of the original referees.

Please attend to all of the reviewers' comments and ensure that you upload both a 'clean' version of your Word file, along with a highlighted version clearly showing where you have made changes in the revised manuscript. Please avoid using 'Tracked changes' in Word files as these are lost in PDF conversion. I should be grateful if you would also provide a point-by-point response detailing how you have dealt with the points raised by the reviewers in the 'Response to Reviewers' box. If you do not agree with any of their criticisms or suggestions please explain clearly why this is so.

Reviewer 1

SUMMARY OF THE ADVANCE MADE IN THIS PAPER AND ITS POTENTIAL SIGNIFICANCE TO THE FIELD

© 2026. Published by The Company of Biologists under the terms of the Creative Commons Attribution License (https://creativecommons.org/licenses/by/4.0/).

The manuscript by Yu & Spradling presents a comprehensive lineage-tracing analysis of Drosophila ovarian follicle development, proposing a revised framework in which three temporally distinct follicle waves arise during pupal stages. In addition to the canonical GSC-derived wave (wave 2), the authors define a direct PGC-derived wave 1.5 responsible for early fertility, and a novel, transient wave 1 population that undergoes rapid proliferation followed by coordinated degeneration and extrusion. Authors went on to show that this germline degeneration and extrusion is also detected in male testes around same time as the large peak of pharate adult ecdysone pulse, which lead authors to propose a causal link between wave 1 follicle degeneration and ecdysone pulse. Furthermore, this study characterized the somatic cell lineage and discovered a novel pupal follicle stem cells (FSC-P) for wave 1.5 follicles and the developmental replacement of FSCs (FSC-P → FSC-A).

The major strength of this manuscript is the clone dataset—well-controlled temporally and quantitatively—allowing the authors to uncover previously unappreciated germ cell populations and stem cell dynamics. The work also provides a compelling new framework for the waves of Drosophila oogenesis, which show an astonishing similarity to mammals. This work thus paves the way to further investigate the functional role of wave 1 follicles and the difference between wave 1.5 and wave 2 follicles. Below are some comments (sequenced based on the importance) to further improve the clarity of the manuscript.

SUGGESTIONS TO AUTHORS

1. The proposed link between wave 1 degeneration and the pharate adult ecdysone pulse is intriguing but remains correlative. No functional perturbation is presented to test whether this population contributes to hormone production/pupal development. Is it possible to prevent wave 1 proliferation or degeneration to see whether ecdysone pulse at 24h APF is disrupted and whether pupal development is stalled?

2. The authors described two classes of FSCs (FSC-A and FSC-P) for wave 2 and wave 1.5 follicle development, respectively. However, the data seem not exclude the model that FSC-P and FSC-A are the same lineage and that the immediate daughter of FSC-P migrate to the niche in the opposite site to replace the FSC-P as it occurs in adult ovary with less frequency.

3. Authors described that "GSC division number stayed constant across the five labeling time points, indicating no GSC division until at least 10h". This interpretation may not be accurate here. According to the data GSC divide about 4 times after 10h APF to adult. It takes more than 12 hour and less than one day to finish one division. The labeling window from -2 to 14h may not allow the GSC to finish one division, thus the total GSC division remain the same, not because no GSC division during this window.

4. While large clone datasets are presented, some of the quantitative methods are not well described in the materials and methods, which made hard for reader to interpret the data and understand the results. For example, Figure 5A, 5E, 5I.

5. Variability across ovarioles and biological replicates is not always clearly presented. The authors can Include more explicit statistical reporting (confidence intervals, variability measures) in some result description. For example, what is the confidence interval for the average number of cysts in the following description: "On average, each ovariole contains 12.5 wave 2 cysts (5 cysts produced per GSC and 2.5 GSCs per ovariole, n=165). Likewise wave 1.5 was responsible for about 6.5 follicles per ovariole (Figure 2D)."

6. "Thus, ~100 wave 1.5 follicles are produced per ovary, a much larger population than previously estimated." How is 100 calculated and what is the assumption here?

7. "Terminal wave 1.5 follicle cell clones frequently contain labeled follicle cells in a posterior patch and less often in other positions (Figure S3G-G')". What is the frequency and sample size, and is it statistically significant?

8. "Initial wave 1.5 follicles also had a higher frequency of germline cyst developmental abnormalities where they contain 8 or 32 cells per follicle (Figure S2H), indicating they underwent

© 2026. Published by The Company of Biologists under the terms of the Creative Commons Attribution License (https://creativecommons.org/licenses/by/4.0/).

one less or one more round of cyst division." There's no data to support the claim of higher frequency.

9. "Averaging all wave 1.5 follicles at a given time point, a steady decline in the percentage of PGC labeling (8-cell clones) was seen as a function of clone induction times, indicating the timing of initial PGC cyst divisions (Figure 2H)." In Figure 2H, it is PGC clones, not 8-cell clones, that decline.

Reviewer 2

SUMMARY OF THE ADVANCE MADE IN THIS PAPER AND ITS POTENTIAL SIGNIFICANCE TO THE FIELD

Here, Fu and Spradling report on a detailed clonal analysis of the Drosophila ovary during development and early adulthood. They identify three waves of follicle formation that closely parallel the three waves, Wave 1, Wave 1.5 and Wave 2 that have been described in mammals. These findings further demonstrate the conservation of ovarian biology between flies and mammals and provide important context for ongoing studies in Drosophila. In general, the data are clearly presented and provide strong support for the conclusions. However, I have a few somewhat minor suggestions for improvement, listed below.

SUGGESTIONS TO AUTHORS

1. Statistical tests should be performed on quantitative data throughout the paper. Statements in the text about quantitative differences (e.g. about the data shown in Figs. 2C, 5A, and 5E) should be supported by appropriate statistical tests of significance. Also, do the lines in Fig. 2H represent least-squared linear regressions? The method of generating those lines should be stated in the figure legend.

2. Please provide further explanation and reasoning for the statement on Page 7 that there is no GSC division until at least 10h. This also comes up on Page 9 with the statement that there is a "Lack of GSC division during this time" though, in this case, the text seems to be referring to 18h ovaries rather than 10 hr ovaries.

3. On page 8, there is a reference to a steady decline in 8-cell clones. Did the authors mean to refer to a steady decline in 16-cell clones?

4. The production of Wave 1.5 follicles directly from PGCs is a very interesting process. Do the authors think that the follicles move in an anterior-posterior direction as they develop or do they develop "in place" without A/P movement? The observation that all four types of Wave 1.5 clones are present simultaneously (regardless of the time of clone induction, as shown in Fig. 2H) indicates that the follicles develop asynchronously. Is the position of the different clone types along the length of the ovariole random or is there a pattern, such as larger clones at more posterior positions, that could suggest A/P movement?

5. Please describe the calculations used to estimate the total number of Wave 1 germ cells that are expelled.

6. Why are there no labeled FSC-A progeny visible in any of the images in Fig. 4 and Figure S2? Is it just due to the selection of images for the figure? The authors may want to comment on this in the figure legend but it is up to them. I do not think that doing so is necessary for publication.

7. Despite all the explanation in the Legends tab, Table S3 is very confusing. Some things I don't understand are listed below

a. If blue = 1 GSC clone, dark blue = more than 1 GSC clone and grey = 0 GSC clones, why aren't all rows one of these three colors? How is it possible to have a row (e.g. Sheet -2h-1, B4) that has no color?

© 2026. Published by The Company of Biologists under the terms of the Creative Commons Attribution License (https://creativecommons.org/licenses/by/4.0/).

b. Box B4 on the Legends tab states: "Numbers in each ovariole row indicate number of clone at the indicated location and size." But, in the example below (G22:J23) all the numbers are zero and yet the description that follows seems to indicate that there is a clone at this position. Do the numbers in each ovariole row only report on the number of germ cell clones (and not follicle cell clones)?
c. In the description of the example in G22:J23, it states that there is 75% coverage on the Stage 4-5 follicle. Is this because three out of four (75%) of the boxes are green? Is there significance to the fact that the one non-green box is in the second position rather than another position in Row 23?
d. What is the significance of the red numbers (e.g. Sheet -2h-1, R4)?
e. How is it possible for there to be a mismatch in the shade of green highlighting individual numbers vs the key at the end (e.g. -2h-1, Row 6 or 2h-1, Row 301 or 6h-1 Rows 42 and 43)? Also, why are there some rows (e.g. 14h-1 Row 16) that do not have a green key at the end?
f. If green indicates follicle cell clones, how can there be green highlighting in the GSC, R1 and R2a columns (e.g. Sheet -2h-1, Rows 75, 265, and 131)?
g. In Column I, what is the difference between an empty cell and a cell with a 0 in it? Also, presumably, an empty cell in columns J through AM just means that stage wasn't present in that ovariole. Is that correct? If so, please add this to the legend.

Reviewer 3

SUMMARY OF THE ADVANCE MADE IN THIS PAPER AND ITS POTENTIAL SIGNIFICANCE TO THE FIELD

In this manuscript, Fu and Spradling describe the use of lineage tracing to characterize three distinct waves of follicle development in the Drosophila ovary. The authors define "wave 2" follicles as those derived from germline stem cells and "wave 1.5" follicles as those formed directly from differentiating PGCs. The presence of both types of these follicles has been noted in the literature, but the authors provide new information regarding both cell numbers within these populations and how these waves contribute to egg production. The authors describe a previously unrecognized third wave, the so called "wave 1" follicles, which undergo programmed cell death and are extruded from the ovary at the beginning of pharate adult formation. A subpopulation of PGCs within testes behaves in a similar manner. The authors speculate that wave 1 germ cells may contribute to the pharate adult ecdysone pulse. The manuscript also provides important insights into the origin and behavior of different somatic cells during pupal development, including two different types of follicle stem cells (FSC-P and FSC-A), escort cells and cap cells. Thus the lineage tracing described in this study provides important insights into a critical period of Drosophila ovary development.

The data generally support the conclusions of the authors and make a significant contribution to our understanding of follicle development in Drosophila. The finding that Drosophila ovaries exhibit three distinct waves of follicle development and that a population of developing germ cells undergoes atresia highlights interesting parallels between flies and other sexually reproducing species, including mammals. Moreover, the presented lineage tracing provides important and novel insights into how different populations of somatic cells support germ cell development during pupal development and in adults. The images and movies presented in the manuscript are beautiful and of very high quality. I believe the manuscript reaches the threshold needed for publication in the journal and that readers of Development will be very interested in this work. That said, several minor points should be addressed before the work is published.

SUGGESTIONS TO AUTHORS

1) Information regarding the staining in various panels throughout the paper (e.g. Figure 3M- likely Vasa, Bam, DNA) is missing.

2) The vacuoles and gaps between cells in Figure 3N,N' are difficult to make out in the presented images. As far as I can tell 3N,N' are images of vasa staining in wave 1 follicles without BODIPY staining. These images should be annotated to highlight the features the authors wish to draw attention too. Do other lipid dyes stain these vacuoles inside cells? Do the authors think these BODIPY positive droplets (shown in Fig 4) are secreted or simply remnants of dying cells as they are being extruded? Further data are needed to fully support the authors' conclusions on these points.

© 2026. Published by The Company of Biologists under the terms of the Creative Commons Attribution License (https://creativecommons.org/licenses/by/4.0/).

3) The authors state "Wave 1 extrusion temporally overlaps with the 24h APF pharate adult ecdysone pulse, along with the observed release of lipid-rich vacuoles, hints at a causal relationship between these events." The idea that these cells may serve as a source of ecdysone is intriguing. The authors are very careful to point out that this idea is speculative. That said, the cause-and-effect relationship between wave 1 cyst death and changes in ecdysone titers is not clear. I worry that the field will adopt this model without sufficient data to support the idea. I realize that additional experiments may be beyond the scope of this paper, but the authors could measure ecdysone titers or reporters of ecdysone signaling and compare control ovaries with mutants that lack germ cells. In addition, recent studies suggest additional sources of ecdysone, including muscle (https://pubmed.ncbi.nlm.nih.gov/38096823/). Is it possible that wave 1 extrusion is in response to increasing ecdysone levels from other sources? Without further data, I suggest removing this speculation from the abstract and further tempering language in the text.

4) Minor: Do the authors observe 8 cell and 32 cell wave 1.5 cysts without heat shock and clone induction? If a general feature of wave 1.5 cysts, one should be able to routinely see these types of cysts in ovaries from young females.

5) Minor: Were all the FSC-P clones shown in Figure 5 marked at 10h APF? If so, this should be stated in the legend or displayed in the figure. The authors should double check that the time of clone induction is provided for all images showing lineage throughout the paper.

First revision

<u>Author response to reviewers' comments</u>

Reviewer 1.

"The work also provides a compelling new framework for the waves of Drosophila oogenesis, which show an astonishing similarity to mammals."

We thank the reviewer for appreciating the importance of our findings about follicle waves.

Below are some comments (sequenced based on the importance) to further improve the clarity of the manuscript.

1. *The proposed link between wave 1 degeneration and the pharate adult ecdysone pulse is intriguing but remains correlative. No functional perturbation is presented to test whether this population contributes to hormone production/pupal development. Is it possible to prevent wave 1 proliferation or degeneration to see whether ecdysone pulse at 24h APF is disrupted and whether pupal development is stalled?*

The purpose of this paper is to establish that Drosophila waves exist and to sufficiently characterize them to allow their function to be studied. The connection of wave 1 to ecdysone production cannot be adequately addressed by any single added experiment. Yes, we have found multiple ways to perturb wave 1 that are associated with disruption of pupal development in most mutant animals. We have been studying this process for nearly two years, but the results are not finished. It will take a full subsequent publication to decisively answer the question you raise and to deal with related issues.

2. *The authors described two classes of FSCs (FSC-A and FSC-P) for wave 2 and wave 1.5 follicle development, respectively. However, the data seem not exclude the model that FSC-P and FSC-A are the same lineage and that the immediate daughter of FSC-P migrate to the niche in the opposite site to replace the FSC-P as it occurs in adult ovary with less frequency.*

Several characteristics of FSC-A clones disprove the possibility that the FSC-A clone labeling pattern can be derived from a canonical adult FSC replacement event. If a labeled FSC-P divides and its

© 2026. Published by The Company of Biologists under the terms of the Creative Commons Attribution License (https://creativecommons.org/licenses/by/4.0/).

daughter cross migrates to replace the unlabeled FSC-P, this would not produce a canonical FSC-A clone. Rather the resulting ovariole would have two labeled FSCs and would start to produce 100% labeled follicles, as commonly occurs in adult ovarioles following FSC replacement (Margolis and Spradling, 1995). If a recombined replacement FSC was activated with the first wave 2 follicle and replaced a labeled FSC-P clone, a very rare event, this would appear as an FSC-P clone that persisted. But at an equal frequency it would replace the unlabeled FSC-P and this again would generate1-2 consecutive 100% labeled follicles by eclosion. 100% labeled follicles were never observed in our experiments.
While we demonstrate that the waves have follicle cells that derive from different stem cells, we do not yet have separate transcriptome data from the FSC-P and FSC-A populations to resolve express different genes. This was exactly the situation in the mouse where BPGs and EPGs were observed to come from different progenitors, but molecular differences associated with cell type rather than just stage of development were not documented until substantially later (Niu and Spradling, 2020).

3. Authors described that "GSC division number stayed constant across the five labeling time points, indicating no GSC division until at least 10h". This interpretation may not be accurate here. According to the data GSC divide about 4 times after 10h APF to adult. It takes more than 12 hour and less than one day to finish one division. The labeling window from -2 to 14h may not allow the GSC to finish one division, thus the total GSC division remain the same, not because no GSC division during this window not because no GSC division during this window.

The reviewer makes a good point that the argument quoted did not fully describe all data leading to the cited conclusion, including the fact it does not depend on lineage data. We revised the manuscript to emphasize on our direct validation of low or absent GSC division before 10h by counting the number and cyst state of all germ cells at the tips of ovarioles in pupal ovaries from -2h to 10h. Staining for 1B1 and bam showed that before 10h there is little if any wave 2 cyst production, nor do transient 2-cell fusomes characteristic of dividing GSCs appear among cap cell-contacting germ cells (potential GSCs), nor do fusomes characteristic of 2-cell cysts arise immediately downstream of these cells, all of which would be expected if GSCs divided earlier. Overall, in ovariolar regions posterior to potential GSCs and anterior to dividing wave 1.5 cysts expressing bam-GFP, there was also no net increase in average germ cell number before 10h.

4. While large clone datasets are presented, some of the quantitative methods are not well described in the materials and methods, which made hard for reader to interpret the data and understand the results. For example, Figure 5A, 5E, 5I.

We were concerned about manuscript length, but have now expanded the Materials and Methods section now that we realize this section is not counted in the 7,000 word limit. aA new section was added "Lineage clone quantifications" that more fully describes data analysis in these Figures panels.

5. Variability across ovarioles and biological replicates is not always clearly presented. The authors can Include more explicit statistical reporting (confidence intervals, variability measures) in some result description. For example, what is the confidence interval for the average number of cysts in the following description: "On average, each ovariole contains 12.5 wave 2 cysts (5 cysts produced per GSC and 2.5 GSCs per ovariole, n=165). Likewise wave 1.5 was responsible for about 6.5 follicles per ovariole (Figure 2D)."

Ovarioles strictly but independently regulate the number of follicles per ovariole in adults using checkpoints, and by regulating the speed of follicle development. As in adults, we did not observe large or interesting variation in cysts or follicles number per pupal ovariole. One can see from the plots of >1,000 ovarioles in Table S3, that ovarioles at eclosion differ very little in follicle or cyst number. We added standard deviation of the numbers mentioned as requested.

6. "Thus, ~100 wave 1.5 follicles are produced per ovary, a much larger population than previously estimated." How is 100 calculated and what is the assumption here?

The number is reached by multiplying the number of wave 1.5 follicles per ovariole (6.4) by the number of ovariole per ovary (15.8) =101.4, this part of the calculation is now added to the text.

© 2026. Published by The Company of Biologists under the terms of the Creative Commons Attribution License (https://creativecommons.org/licenses/by/4.0/).

7. "Terminal wave 1.5 follicle cell clones frequently contain labeled follicle cells in a posterior patch and less often in other positions (Figure S3G-G')". What is the frequency and sample size, and is it statistically significant?

This information was added to the results section.

8. "Initial wave 1.5 follicles also had a higher frequency of germline cyst developmental abnormalities where they contain 8 or 32 cells per follicle (Figure S2H), indicating they underwent one less or one more round of cyst division. "There's no data to support the claim of higher frequency .

The complete distribution of follicles with 8 or 32 follicles by position can be found in Table S3 by cells with orange shading. We added data, and N values, percentages and a statistical test supporting the significance of our statement, where "initial" actually means the most mature follicle in the ovariole that will be laid first.

9. "Averaging all wave 1.5 follicles at a given time point, a steady decline in the percentage of PGC labeling (8-cell clones) was seen as a function of clone induction times, indicating the timing of initial PGC cyst divisions (Figure 2H). "In Figure 2H, it is PGC clones, not 8-cell clones, that decline.

This is a common misconception inherent to this labeling system. 8-cell clones are generated by recombination in PGCs that commit to cyst formation and segregate a labeled and unlabeled daughter at the first division. This makes them equivalent to CBs as explained in Figure 1E, and they are the source of nearly all w1.5 cysts. 16-cell labeled wave 1.5 cysts are only caused by rare PGC symmetric divisions and while they are fully documented in Table S3 and an example shown in Figure 2F, they are not plotted in Figure 2H.

Reviewer 2:

"In general, the data are clearly presented and provide strong support for the conclusions. However, I have a few somewhat minor suggestions for improvement, listed below."

We thank the reviewer for appreciating the strength of our conclusions, and for their suggestions for improvement, which we address point by point below.

1. Statistical tests should be performed on quantitative data throughout the paper. Statements in the text about quantitative differences (e.g. about the data shown in Figs. 2C, 5A, and 5E) should be supported by appropriate statistical tests of significance. Also, do the lines in Fig. 2H represent least-squared linear regressions? The method of generating those lines should be stated in the figure legend.

We have performed the statistical tests to bolster our statements of quantitative difference as suggested and included them in figure legends to avoid cluttering of the figures. Yes the lines in Figure 2H were drawn by a program using least squares, and we added a new Materials and Methods section explaining the derivation of Figure 2C, 2H, 5A, 5E in more detail.

2. *Please provide further explanation and reasoning for the statement on Page 7 that there is no GSC division until at least 10h. This also comes up on Page 9 with the statement that there is a "Lack of GSC division during this time" though, in this case, the text seems to be referring to 18h ovaries rather than 10 hr ovaries.*

The GSCs marked at earlier time points had more time to develop and proliferate, since they had more time between HS label activation and analysis (all flies were dissected exactly 5 days after pupariation or ~12 hours after eclosion, so if HS is administered earlier, the labeled cells have more time to develop before analysis). Yet the number of progeny made by GSCs stayed constant from -2h until 14h (Figure 2C yellow line) even as the GSCs labeled at -2h had 16 more hours to proliferate than those labeled at 14h, indicating they underwent minimal to no division during this

© 2026. Published by The Company of Biologists under the terms of the Creative Commons Attribution License (https://creativecommons.org/licenses/by/4.0/).

time. We changed the 10h in the text to 14h to better reflect this idea, we originally proposed it to be 10h since blue line in Figure 2C shows a GSC population expansion by 10h and that could mark the starting point of GSC proliferation, but that is more likely due to GSC niche expansion and more PGCs getting into contact with the niche than being caused by GSC proliferation. We have also changed the text on page 9 to more clearly refer to this period of time. In addition we directly observed GSCs in pupal ovaries stained with Hts to reveal fusomes. They retained characteristic round fusomes and if GSC were dividing we would have detected distinctive fusome changes associated with asymmetric GSC division as explained in the answer to reviewer 1 point 3.

3. On page 8, there is a reference to a steady decline in 8-cell clones. Did the authors mean to refer to a steady decline in 16-cell clones?

Because of the nature of the clonal marking system used, 8-cell clones represent PGCs that directly differentiated. See response for comment 9 of reviewer 1 for detailed explanation of this point.

4. The production of Wave 1.5 follicles directly from PGCs is a very interesting process. Do the authors think that the follicles move in an anterior-posterior direction as they develop or do they develop "in place" without A/P movement? The observation that all four types of Wave 1.5 clones are present simultaneously (regardless of the time of clone induction, as shown in Fig. 2H) indicates that the follicles develop asynchronously. Is the position of the different clone types along the length of the ovariole random or is there a pattern, such as larger clones at more posterior positions, that could suggest A/P movement?

Yes, wave 1.5 follicles move sequentially within the ovariole between the times we labeled and eclosion. Most of them become the follicles outside the germarium which span a much larger linear dimension than the germarium. Clearly the divisions are not synchronous, and there is no evidence of the last division before 14h (Figure 2H, 8-cell cyst). Figure 1H is an average of all the wave 1.5 cyst data at each time point, converted to % of wave 1.5 follicles, so that all time points can be compared. Recombination indelibly marks the cyst division stage at the time of heat shock, and we can read this out days later in newly eclosed ovaries, regardless of possible early delays while wave 1 completes its program. We have observed that for the direct-differentiating PGCs, a more posterior position in the ovariole is correlated with a later cyst division stage at any given time. For example, the patterns characteristic of later cyst divisions- i.e, 1 cell and 2 cell clones, are absent in the youngest follicles, and the frequency increases uniformly as the follicles become more developed by eclosion (see Table S3, Fig. 2H; Figure S1D-E). This is shown by both characterizing cyst lineage clone size and directly visualizing cyst size in the developing pupal ovary.

5. Please describe the calculations used to estimate the total number of Wave 1 germ cells that are expelled.

Figures S2D-S2E show quantification of the total number of cysts in Wave 1, which reaches a maximum of 21.7 cell clusters on average at 18h and starts to decrease later due to extrusion. However, the number of cells in a cluster increases due to ongoing cyst division. Our data suggests most or all cysts continue cyst division to reach the final 16-cell size. For example, at 26h 72.4% of all observed Wave 1 clusters are 16-cell cysts (Figure 4D, blue line). Fusome staining of cysts at the broken ovary edge or following extrusion were always 16-cell when they could be visualized. So if all independent wave 1 clusters, 21.7, do reach 16 the upper limit of wave 1 cells would be 21.7x16 = 347. However, we cannot rule out that some smaller cysts are also extruded despite failed to observe that. If only 72.4% of 21.7 Wave 1 clusters reach 16-cell size eventually, that would be 251 germ cells. Adding in the remaining 27.6% of Wave 1 cysts that are at smaller cyst sizes based on the proportions observed at 22h at 8CC:4CC:2CC=7:4:4 (Figure S2E), that would bring the overall total Wave 1 population to an average of 282 germ cells. Our previous estimation of 250-350 was intended to span the range of these estimates, but the most likely estimate with current knowledge would be close to the midpoint.

6. Why are there no labeled FSC-A progeny visible in any of the images in Fig. 4 and Figure S2? Is it just due to the selection of images for the figure? The authors may want to comment on this in the figure legend but it is up to them. I do not think that doing so is necessary for publication.

© 2026. Published by The Company of Biologists under the terms of the Creative Commons Attribution License (https://creativecommons.org/licenses/by/4.0/).

Figure 4 shows pupal ovaries where HS was administered 24 hours before analysis, it was not enough time for the labeled FSCs to produce a clone big enough from which we can determine whether it is an FSC-A or FSC-P. We did label FSC-like cells that were observed in such pupal ovaries, but they at most have only produced a clone of 4 cells. Since FSC clones labeled and observed in pupal ovaries were inconclusive, we did not show such clones in the figures.

Images in Figure S2 were also meant to demonstrate cells other than FSC-A clones; these images happened to not contain FSC-A clones as well, so that no FSC-A clones were visible in those images. We recorded images of nearly all the clones listed in Table S3, and 3D movies of many.

7. Despite all the explanation in the Legends tab, Table S3 is very confusing. Some things I don't understand are listed below

a. If blue = 1 GSC clone, dark blue = more than 1 GSC clone and grey = 0 GSC clones, why aren't all rows one of these three colors? How is it possible to have a row (e.g. Sheet -2h-1, B4) that has no color?

Grey = 0 germ cell clones, including both wave 2 (GSC, CB) clones and wave 1.5 clones. We have now more clearly stated this definition in the legends.

b. Box B4 on the Legends tab states: "Numbers in each ovariole row indicate number of clone at the indicated location and size." But, in the example below (G22:J23) all the numbers are zero and yet the description that follows seems to indicate that there is a clone at this position. Do the numbers in each ovariole row only report on the number of germ cell clones (and not follicle cell clones)?

Yes that is correct, numbers indicate only germ cell clones while somatic cell clones are marked by green shading.

c. In the description of the example in G22:J23, it states that there is 75% coverage on the Stage 4-5 follicle. Is this because three out of four (75%) of the boxes are green? Is there significance to the fact that the one non-green box is in the second position rather than another position in Row 23?

Yes, 3 out of 4 cells being green means a 75% labeling rate. The relative location of the unlabeled cell also indicates where the unlabeled follicle cell patch exist on the actual follicle. In the example given in the legend: means a small patch in the mid-anterior region of this follicle is unlabeled.

0	0	0	0

d. What is the significance of the red numbers (e.g. Sheet -2h-1, R4)?

W1.5 germ cell clones are marked in red (1) for easier spotting among the 0s. We updated the legends to clarify this point.

e. How is it possible for there to be a mismatch in the shade of green highlighting individual numbers vs the key at the end (e.g. -2h-1, Row 6 or 2h-1, Row 301 or 6h-1 Rows 42 and 43)? Also, why are there some rows (e.g. 14h-1 Row 16) that do not have a green key at the end?

We checked the rows mentioned and did not find a mismatch. However, this prompted us to go over the table more carefully and we indeed found a few mismatches elsewhere. We thank the reviewer for carefully going over the table.

f. If green indicates follicle cell clones, how can there be green highlighting in the GSC, R1 and R2a columns (e.g. Sheet -2h-1, Rows 75, 265, and 131)?

Those would represent TF, CC and EC clones. We updated the legends to better clarify this point.

© 2026. Published by The Company of Biologists under the terms of the Creative Commons Attribution License (https://creativecommons.org/licenses/by/4.0/).

g. In Column I, what is the difference between an empty cell and a cell with a 0 in it? Also, presumably, an empty cell in columns J through AM just means that stage wasn't present in that ovariole. Is that correct? If so, please add this to the legend.

That is correct, and we have added this point to the legends.

Reviewer 3:

"The data generally support the conclusions of the authors and make a significant contribution to our understanding of follicle development in Drosophila. The finding that Drosophila ovaries exhibit three distinct waves of follicle development and that a population of developing germ cells undergoes atresia highlights interesting parallels between flies and other sexually reproducing species, including mammals. Moreover, the presented lineage tracing provides important and novel insights into how different populations of somatic cells support germ cell development during pupal development and in adults. The images and movies presented in the manuscript are beautiful and of very high quality. I believe the manuscript reaches the threshold needed for publication in the journal and that readers of Development will be very interested in this work. That said, several minor points should be addressed before the work is published."

We thank the reviewer for appreciating the significance of our findings. We have attempted to address their suggestions as indicated below:

1) Information regarding the staining in various panels throughout the paper (e.g. Figure 3M- likely Vasa, Bam, DNA) is missing.

The legend to Figure 3 states that the same staining shown in panel B, i.e. bam, Hts (1B1), Vas, DAPI, is also used in panels B-J We rely on this common mechanism because of space limitation. The reviewer is correct that staining in L, Hts (1B1), Vas, DAPI should have been extended to panels M and N. This was an oversight that is now fixed.

2) The vacuoles and gaps between cells in Figure 3N,N' are difficult to make out in the presented images. As far as I can tell 3N,N' are images of vasa staining in wave 1 follicles without BODIPY staining. These images should be annotated to highlight the features the authors wish to draw attention too. Do other lipid dyes stain these vacuoles inside cells? Do the authors think these BODIPY positive droplets (shown in Fig 4) are secreted or simply remnants of dying cells as they are being extruded? Further data are needed to fully support the authors' conclusions on these points.

The reviewer is correct that 3N and N' are stained with Vasa and Hts (1B1). There was no Bodipy staining used here. We took the reviewer's suggestion and added arrows to indicate the internal vacuoles in N and N'. While the images are small (due to the number of figure panels), they contain additional detail; the images can be magnified in the pdf version of the article, revealing this information. We have stained these vacuoles in many independent experiments in males and females using Bodipy dye 493/503 which is selective for neutral lipids). We think that the germ cells interact with somatic cells, possibly fusing to make the very large cells pictured in brightfield in Figure 4H' and I' before breaking open to release the vesicles and cellular debris into the hemolymph. We have a lot of additional data, but it will require additional more experiments to resolve remaining uncertainties and provide further data.

3) The authors state "Wave 1 extrusion temporally overlaps with the 24h APF pharate adult ecdysone pulse, along with the observed release of lipid-rich vacuoles, hints at a causal relationship between these events." The idea that these cells may serve as a source of ecdysone is intriguing. The authors are very careful to point out that this idea is speculative. That said, the cause-and-effect relationship between wave 1 cyst death and changes in ecdysone titers is not clear. I worry that the field will adopt this model without sufficient data to support the idea. I realize that additional experiments may be beyond the scope of this paper, but the authors could measure ecdysone titers or reporters of ecdysone signaling and compare control ovaries with mutants that lack germ cells. In addition, recent studies suggest additional sources of ecdysone,

© 2026. Published by The Company of Biologists under the terms of the Creative Commons Attribution License (https://creativecommons.org/licenses/by/4.0/).

including muscle (https://pubmed.ncbi.nlm.nih.gov/38096823/). Is it possible that wave 1 extrusion is in response to increasing ecdysone levels from other sources? Without further data, I suggest removing this speculation from the abstract and further tempering language in the text.

We have carried out additional studies as described in our response to reviewer 1, comment 1. We have already been careful not to say that ecdysone is being released from the germ cells, but only that the extrusion is involved in the production of the pharate adult pulse in some way. The vacuole could provide other lipids or signals involved, rather than the hormone itself. We have no prejudice against other tissues making a contribution; for example, several tissues are known to contribute to yolk protein production in adults. But we do not believe wave 1 turnover is due to ecdysone-mediated apoptosis for reasons stated in the Discussion. Other tissues that turn over as a result of ecdysone as part of metamorphosis, do not undergo a massive expansion in cell number and coordinate with a population of somatic (swarm) cells that migrate to the site of turnover days earlier during late L3. We are confident this is a programmed developmental process. Mouse researchers dismissed wave 1 remodeling as "atresia" for decades, thereby missing its significance in steroid hormone production.

4) Minor: Do the authors observe 8 cell and 32 cell wave 1.5 cysts without heat shock and clone induction? If a general feature of wave 1.5 cysts, one should be able to routinely see these types of cysts in ovaries from young females.

We did not study this process without heat shock, but heat shock has been widely used in studies of the adult ovary without causing such effects on cyst formation. My lab has carried out many studies of heat shock including with this lineage labeling system (Margolis and Spradling, 1995) on Drosophila ovaries without seeing an effect like this. The most posterior wave 1 follicles are forced to develop unusually fast to produce follicles in time for eclosion and preferentially show the effect. It is probably not easy to study these follicles in young adult females, as the reviewer suggests, because cysts with the wrong number of nurse cells fail to generate fertile oocytes. Less damaging defects may also occur, and looking for evidence of differential fitness of laid eggs from this wave under different conditions represent follow up studies of wave 1.5 that should be addressed in the future.

5) Minor: Were all the FSC-P clones shown in Figure 5 marked at 10h APF? If so, this should be stated in the legend or displayed in the figure. The authors should double check that the time of clone induction is provided for all images showing lineage throughout the paper.

No, FSC-P clones come from multiple times, and we saw no pattern differences. Labeling induction times for every clone pictured would clutter the manuscript with irrelevant information. The comparative information that used timing is already shown in many graphs in the manuscript.

Second decision letter

MS ID#: dev.205668R1

MS TITLE: The Drosophila ovary produces two waves of adult follicles and a novel pupal wave that turns over

AUTHORS: Yunpeng Wayne Fu and Allan Spradling

Dear Dr Spradling,

I have now received all the referees reports on the above manuscript, and have reached a decision. The referees' comments are appended below.

The overall evaluation is positive and we would like to publish a revised manuscript in Development. Reviewer 1 makes suggestions to align some numbers in the text with the figures and

© 2026. Published by The Company of Biologists under the terms of the Creative Commons Attribution License (https://creativecommons.org/licenses/by/4.0/).

other textual changes. I ask that you address these changes for clarity, and rigor. While I do not expect to send the manuscript back to the reviewer, I will look at these myself, thus I ask that you attend to all of the reviewers' comments in your revised manuscript and detail them in your point-by-point response. If you do not agree with any of their suggestions explain clearly why this is so.

Reviewer 1

SUMMARY OF THE ADVANCE MADE IN THIS PAPER AND ITS POTENTIAL SIGNIFICANCE TO THE FIELD

The manuscript by Yu & Spradling characterized the three waves of follicle development in Drosophila, which is reminiscent to the recent discoveries in mammalian system. The discovery of new Wave 1 follicles in pupal development is very interesting and sets the foundation for characterization of their significance for animal development and reproduction. The coordinated replacement of pupal FSCs by the adult FSCs is also a novel discovery and likely provide a new model for study stem cell behavior.

SUGGESTIONS TO AUTHORS

The revised manuscript by Yu and Spradling is significantly improved in terms of clarification and statistic analysis. It addressed most of the reviewers' comments. I don't have any major concerns but a minor suggestion for the author below.

The number of ovarioles, GSC clone, and GSC clone frequency in Figure 2C, Table S1, and Table S3 is slightly inconsistent. In the text, it mentioned 388 ovarioles from 10-14h and 1579 ovarioles from three earlier times. In the Table S1, it showed 778 (346+303+129) ovarioles, not 1579, from three earlier times. In Table S1 lower table, the frequency of GSC at 14h is 28.3%. However, the upper table showed the number as 45 out of 164 (27.4%, not 28.3%) at 14h. Furthermore, Table S3 only showed 160 ovarioles for 14h, not 164. The total number of ovarioles with GSC clones for 14h is 38 not 45. According to Table S3, the frequency for GSC clones at 14h is around 24%. Please double check the number to make it accurate.

Reviewer 2

With these revisions, the authors have fully addressed my concerns and I am now supportive of publication.

Reviewer 3

The authors have addressed all of my previous concerns. I find the paper acceptable for publication.

Second revision

Author response to reviewers' comments

We thank the reviewer for carefully going over our data. Following are the responses to the reviewer’s questions:

The number of ovarioles, GSC clone, and GSC clone frequency in Figure 2C, Table S1, and Table S3 is slightly inconsistent. In the text, it mentioned 388 ovarioles from 10-14h and 1579 ovarioles from three earlier times. In the Table S1, it showed 778 (346+303+129) ovarioles, not 1579, from three earlier times.

© 2026. Published by The Company of Biologists under the terms of the Creative Commons Attribution License (https://creativecommons.org/licenses/by/4.0/).

We corrected the final ovariole number from 1,579 to 768 based on the final version of Table S1. The original larger number was taken from an earlier version that included additional replicates whose scoring was not finished, after we realized that this larger number was unnecessary for the project.

In Table S1 lower table, the frequency of GSC at 14h is 28.3%. However, the upper table showed the number as 45 out of 164 (27.4%, not 28.3%) at 14h. Furthermore, Table S3 only showed 160 ovarioles for 14h, not 164. The total number of ovarioles with GSC clones for 14h is 38 not 45. According to Table S3, the frequency for GSC clones at 14h is around 24%. Please double check the number to make it accurate.

We fixed all of these minor issues. The number of ovarioles at 14h as the reviewer noted was changed to 160 from 164. We had mistakenly taken the larger number from an earlier Table version. However, the total number of GSC at 14h is indeed 45 as we reported. The reviewer was correct that the number of ovarioles containing GSC is 38, but some ovarioles contain more than 1 labeled GSC, resulting in a larger number of total labeled GSCs. Using these corrected numbers resolves the discrepancy in the GSC labeling frequencies (27.4% vs the correct 28.3%) noted by the reviewer as well.

Third decision letter

MS ID#: dev.205668R2

MS TITLE: The Drosophila ovary produces two waves of adult follicles and a novel pupal wave that turns over

AUTHORS: Yunpeng Wayne Fu and Allan Spradling

Dear Allan,

I am happy to tell you that your manuscript has been accepted for publication in Development, pending our standard publication integrity checks.

© 2026. Published by The Company of Biologists under the terms of the Creative Commons Attribution License (https://creativecommons.org/licenses/by/4.0/).
